# Research on State Recognition in Aircraft Skin Laser Paint Stripping Based on the Fusion of LIBS Spectra and Surface Images

**DOI:** 10.3390/s26103162

**Published:** 2026-05-16

**Authors:** Haijie Hua, Yongbo Wang, Tian Tan, Shaolong Li, Yu Cao, Zhongxian Tan, Junchao Li, Wenfeng Yang

**Affiliations:** 1 College of Aviation Engineering, Civil Aviation Flight University of China, Guanghan 618307, China; 2 Intelligent Manufacturing Institute of Laser and Optoelectronic, Wenzhou University, Wenzhou 325035, China

**Keywords:** laser-induced breakdown spectroscopy (LIBS), surface imaging, multimodal fusion, laser paint stripping, state recognition

## Abstract

To address the recognition challenges caused by blurred state boundaries and the limitations of single monitoring modalities during aircraft skin laser paint stripping, this study proposes a multimodal data fusion method for state recognition based on laser-induced breakdown spectroscopy (LIBS) and surface imaging. By constructing a synchronous monitoring platform, a dataset covering five key physical states, namely topcoat (Tc), topcoat–primer transition (Tc-Pr), primer (Pr), primer–substrate transition (Pr-As), and substrate damage (As), was established. The proposed gated weighted multimodal fusion network (PGMF-Net) employs SE-ResNet1D to capture variations in elemental composition features from the spectra and integrates ResNet18 to extract changes in surface morphology from the images. The experimental results show that the proposed model outperforms the single-modal methods as well as the compared early-fusion and late-fusion methods, achieving a recognition accuracy of 94.12% on the test set and an average accuracy of 94.87% in stratified cross-validation. The bootstrap-based confidence interval analysis further verifies the stability of this method under the current dataset conditions. Further analysis indicates that the single-spectrum model has difficulty effectively distinguishing coating transition states because different transition states contain identical or highly similar characteristic peak information. The single-vision model, however, shows insufficient sensitivity to subtle substrate damage, whereas multimodal fusion enables complementary representation of material composition information and surface morphological information. Experimental validation under different power conditions further confirms that the model outputs are generally consistent with the macroscopic morphological evolution observed on the sample surface. This method compensates for the limitations of traditional single-source monitoring and provides a methodological foundation for online monitoring and state feedback during the laser paint stripping process.

## 1. Introduction

As aircraft service life increases, surface coatings on aircraft skin are prone to aging, cracking, and peeling under the combined effects of temperature–humidity cycling, mechanical wear, and complex service environments. During maintenance, the removal of the old coating is generally required before substrate defect inspection and repainting can be carried out. Compared with mechanical grinding and chemical paint stripping, laser paint removal offers advantages such as non-contact operation, high selectivity, low pollution, and ease of automated integration. It has therefore demonstrated promising application prospects in the maintenance of aluminum alloy and composite material surfaces and is regarded as a highly promising green alternative technology in aviation maintenance [1,2,3,4].

However, laser paint stripping is not a simple surface removal process, but rather a highly nonlinear physical process jointly influenced by laser energy density, scanning speed, pulse parameters [5], material thermophysical properties, and the coupled interactions at multilayer coating interfaces [6,7,8]. For multilayer coating systems such as those on aircraft skin, the paint stripping process is often accompanied by the coexistence of multiple mechanisms, including thermal decomposition, evaporation, delamination, and plasma-induced impact effects [9,10]. When process parameters are improperly selected, problems such as residual coating, over-cleaning, oxidation caused by heat accumulation, and even substrate damage are very likely to occur [11]. Therefore, the core bottleneck of laser paint stripping lies in achieving stable and controllable state regulation during the removal process [12,13].

To improve the controllability of the laser paint stripping process, a variety of online monitoring approaches have been investigated both domestically and internationally, with typical methods including acoustic monitoring, visual monitoring, and spectroscopic monitoring. Studies have shown that acoustic emission or photoacoustic signals can reflect stage-wise changes in the ablation process and can be used to determine cleaning thresholds and potential damage; however, such signals are essentially indirect representations and are highly susceptible to environmental noise, operating condition fluctuations, and signal interpretation methods [14,15]. Visual monitoring can more intuitively reflect changes in flame morphology, surface color, texture roughness, and cleaning area boundaries, and in recent years has also been combined with deep learning for laser cleaning quality evaluation and surface roughness prediction; however, it mainly relies on macroscopic morphological information and lacks the ability to directly perceive changes in material composition and interfacial elements [16]. In contrast, LIBS offers advantages such as real-time response, high speed, and elemental sensitivity, and has been applied to online monitoring and two-dimensional mapping analysis of laser cleaning processes [17,18]. However, for samples with similar elemental compositions, weak differences between adjacent states, or blurred transition interfaces, recognition based solely on spectral information still faces considerable challenges. In recent years, based on the above single-modal monitoring approaches, a number of studies have conducted specific explorations in the analysis of visual, spectral, and acoustic signals, and have preliminarily verified their feasibility for monitoring laser cleaning/paint stripping processes. Hu et al. [19] proposed a deep learning-based visual process monitoring method for aircraft coating laser cleaning, which was used for cleaning quality and surface state evaluation. Wang et al. [20] introduced LIBS into the online quality monitoring of CFRP laser cleaning and verified the feasibility of spectral information for discriminating cleaning states. Choi et al. [21,22] employed CNNs to identify LIBS spectra and carried out laser paint stripping process monitoring based on multivariate correlation analysis, further demonstrating the advantages of spectroscopic methods in characterizing material composition. For aircraft skin applications, Yang et al. [23] and Li et al. [24] further conducted studies on LIBS-based online thickness prediction and monitoring criteria. Overall, although existing studies have provided a certain foundation for laser cleaning monitoring, they still mainly focus on single-modal monitoring and evaluation based on a single quality index. Research on state recognition for large-area continuous laser paint stripping of aircraft skin that simultaneously integrates LIBS spectra sensitive to material composition and visual images sensitive to surface morphology, while addressing multilayer coating layer-by-layer removal and transition interface identification, remains relatively insufficient.

Against this background, deep learning has provided a new technical pathway for multimodal data fusion. Baltrušaitis et al. [25] systematically reviewed the core issues in multimodal machine learning and pointed out that representation, alignment, fusion, and co-learning are the key components of multimodal modeling. Tang et al. [26] and Zhao et al. [27] further summarized the development trajectory of multimodal sensor fusion and deep multimodal data fusion from the perspective of deep learning, indicating that deep models can automatically learn hierarchical representations from heterogeneous data and explicitly model the complementary relationships among modalities at either the feature level or the decision level. Meanwhile, the cross-modal attention modeling approach proposed by Tsai et al. [28] and the gated fusion strategy introduced by Zhang et al. [29] further demonstrate that attention mechanisms, gating mechanisms, and adaptive weighting strategies have shown potential advantages in handling modal inconsistency, noise disturbances, and cross-modal collaboration.

On this basis, targeting the engineering scenario of large-area laser paint stripping on aircraft skin, this study developed a multimodal monitoring platform capable of synchronously acquiring LIBS spectra and surface images, and divided the paint stripping process into five states: topcoat (Tc), topcoat–primer transition (Tc-Pr), primer (Pr), primer–substrate transition (Pr-As), and substrate damage (As). Based on this framework, a multimodal dataset for laser paint stripping state recognition was established, and a gated weighted multimodal recognition method integrating spectral compositional information with surface morphological information was proposed to improve state discrimination capability under complex operating conditions. Furthermore, through model training and testing, stratified cross-validation, and paint stripping experiments under different power conditions, the recognition performance, stability, and engineering applicability of the proposed method were validated, thereby providing a methodological basis for online monitoring, state feedback, and subsequent intelligent control research in the laser paint stripping process.

## 2. Experimental Design

### 2.1. Experimental Materials and Apparatus

**Figure 1:**
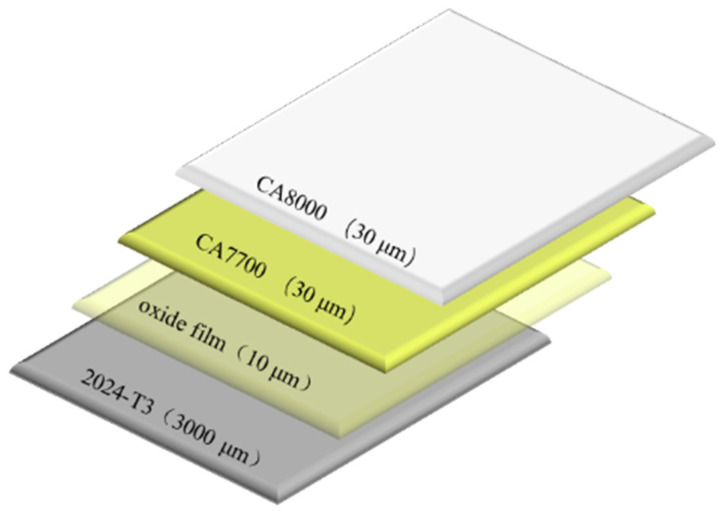
Schematic illustration of coating layer distribution.

The materials investigated in this study were prepared in accordance with the requirements of the A320 Structural Repair Manual [30] and mainly consisted of an aluminum alloy substrate and a surface coating system. To ensure that the material preparation process complied with the specification requirements for civil aircraft skin, both the surface treatment of the aluminum alloy substrate and the coating application were carried out by the airline. A schematic illustration of the coating layer distribution is shown in Figure 1.

To prepare a sample dataset representing different paint removal states of aircraft skin, an infrared pulsed fiber flat-top laser with a wavelength of 1064 nm (Raycus RFL-P1000H RFL-P1000H, Wuhan Raycus Fiber Laser Technologies Co., Ltd., Wuhan, China) was employed in this experiment as the cleaning source. Featuring stable energy output and a wide adjustable range of processing parameters, this laser enables precise control over the layer-by-layer removal of the coating. By adjusting parameters such as laser power and scanning speed, the dynamic evolution process from topcoat removal to primer exposure and subsequently to microscopic substrate damage can be reproducibly achieved, thereby providing high-quality and well-characterized experimental data to support the development of the subsequent multimodal state recognition model.

The laser cleaning experimental setup developed in this study mainly consists of a pulsed fiber laser, a galvanometer scanning system, an industrial robot motion platform, a computer control system, and an online monitoring unit. The laser beam performs high-speed line scanning through the galvanometer system, while the integrated cleaning head is mounted at the end of a robotic arm (KR 70 R2100, KUKA, Augsburg, Germany) and, driven by the robot, accomplishes area scanning and cleaning along a predefined path. The computer control system is used to uniformly set process parameters such as laser power, repetition frequency, scanning speed, and overlap ratio, while also enabling coordinated control of laser output and robotic arm motion. This setup combines local high-precision scanning with large-area continuous cleaning capability, and can effectively simulate the actual paint removal conditions of complex curved aircraft skin components. To meet the requirements of subsequent paint removal state recognition research, the experimental platform was synchronously integrated with LIBS spectral acquisition and surface image capture modules, enabling the recording of spectral responses and surface visual features at different stages during the cleaning process and providing data support for the development of a multimodal deep learning model.

**Figure 2:**
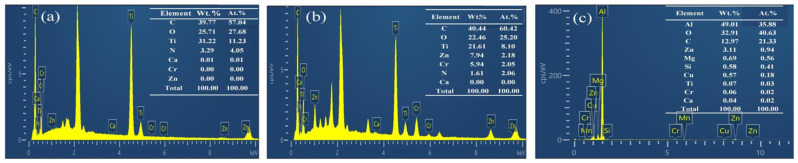
EDS results of the topcoat, primer, and aluminum alloy substrate: (**a**) topcoat; (**b**) primer; (**c**) aluminum alloy substrate.

To clarify the compositional differences among multilayer materials and to identify effective monitoring features, energy-dispersive spectroscopy (EDS) was first performed on the aluminum alloy substrate (2024-T3), primer (CA7700, PPG, Pittsburgh, PA, USA), and topcoat (CA8000, PPG, Pittsburgh, PA, USA), and the results are shown in Figure 2. As can be seen from the figure, significant differences exist in the elemental composition of each layer: the topcoat is mainly enriched in C, O, and the characteristic element Ti; the primer contains not only Ti but also the characteristic elements Zn and Cr; whereas the aluminum alloy substrate is dominated by Al, accompanied by small amounts of Mg and Cu. Variations in the concentrations of these layer-specific elements, such as Ti in the topcoat, Zn and Cr in the primer, and Al in the substrate, constitute the physical basis for state recognition during laser paint stripping. Accordingly, by consulting the Atomic Spectra Database (ASD) of the National Institute of Standards and Technology (NIST), it was found that the characteristic emission lines of the above key metallic elements are mainly concentrated in the visible to near-ultraviolet spectral range. Considering spectral intensity, freedom from interference peaks, and repeatability, this study ultimately determined the monitoring wavelength range to be 336–700 nm. Based on these requirements, BZ2000 and BZ4000 spectrometer modules from Shanghai Fuxiang Optics Co., Ltd., Shanghai, China were finally selected to construct the spectral monitoring system, and their basic parameters are listed in Table 1. This system is capable of fully covering the acquisition requirements for the characteristic spectral lines of the key elements mentioned above.

**Figure 3:**
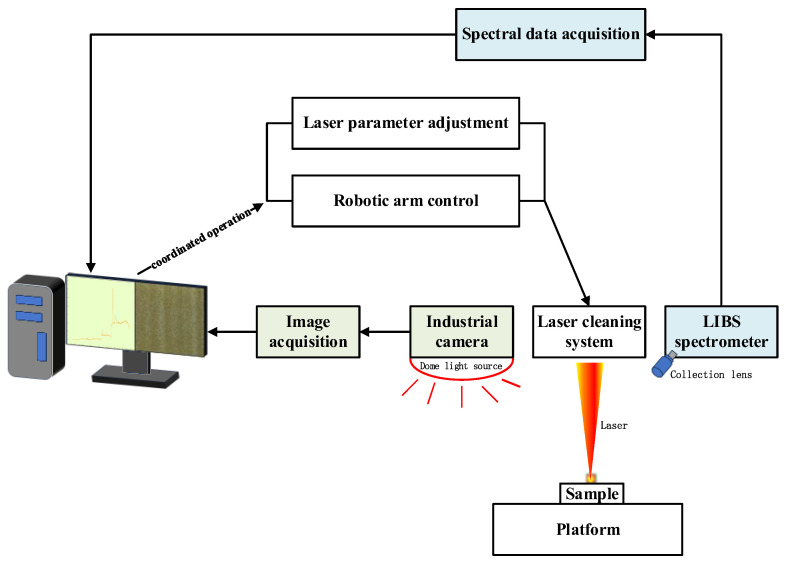
Schematic diagram of experimental setup.

The visual monitoring platform mainly consisted of an industrial area-scan camera (MV-CS060-10GC, HIKROBOT, Hangzhou, China) and an imaging lens (MVL-KF1228M-12MP, 12 mm focal length, HIKROBOT, Hangzhou, China), an auxiliary light source, a camera mounting bracket, and an image acquisition terminal. The overall structure of the experimental setup is shown in Figure 3.

**Figure 4:**
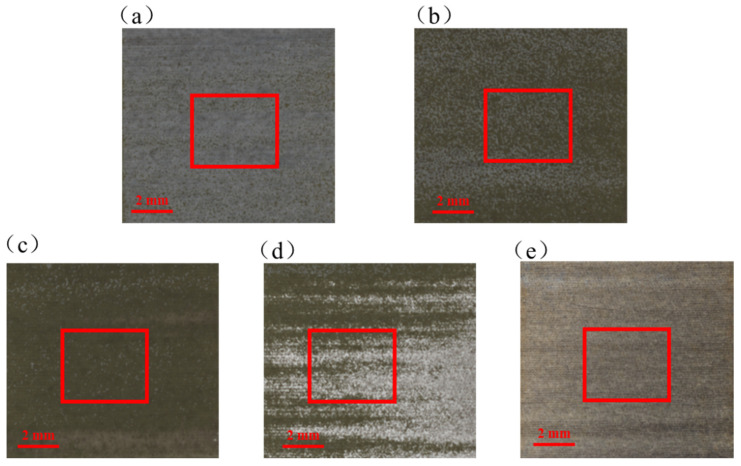
Classification of laser paint stripping results: (**a**) Tc; (**b**) Tc-Pr; (**c**) Pr; (**d**) Pr-As; (**e**) As.

**Figure 5:**
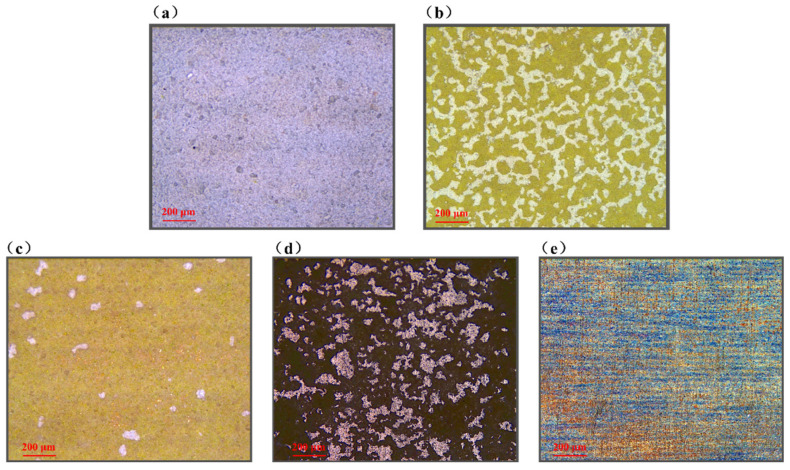
Microscopic images of laser paint stripping classification corresponding to the red boxed regions in Figure 4: (**a**) Tc; (**b**) Tc-Pr; (**c**) Pr; (**d**) Pr-As; (**e**) As.

To achieve accurate recognition of states during the laser paint stripping process, multimodal data acquisition and dataset construction were further carried out after establishing the visual monitoring platform and the LIBS spectral monitoring platform. First, cleaning regions with different degrees of removal were prepared on the sample surface by varying the laser processing parameters. Subsequently, surface images and LIBS spectra were synchronously acquired from the same region using the monitoring platform, and one image and one reconstructed spectrum with strictly corresponding spatial positions were combined into one multimodal data pair as an independent training sample. In practical online monitoring, the model uses only macroscopic images (as shown in Figure 4) and spectra as joint inputs. However, to ensure the absolute scientific validity of the data labels, additional microscopic observations (as shown in Figure 5) were introduced in this study as physical calibration. Accordingly, the paint stripping process was divided into five states. The Tc state is characterized by continuous topcoat coverage, a uniform macroscopic appearance, and intact microscopic texture without exposure of heterogeneous phases. The Tc-Pr state is characterized by partial removal of the topcoat and the initial exposure of the primer, with microscopic coexistence of the residual topcoat and exposed primer. In the Pr state, the white topcoat has been almost completely removed on the macroscopic scale, with only extremely sparse residues remaining under microscopic observation, and the overall surface visual features tend to become uniform. The Pr–As state is characterized by the alternating distribution of residual primer and initial substrate exposure, showing evident interlayer transition features. The As state is dominated by substrate exposure, accompanied by surface roughening, scratches, or over-cleaning marks. This classification scheme can effectively reflect the surface evolution behavior during laser paint stripping, from the layer-by-layer removal of the coating to substrate exposure and damage formation, thereby providing a clear labeling basis for the subsequent development of the multimodal recognition model. Finally, the calibrated multimodal dataset was divided into training, validation, and test sets at a ratio of 7:1.5:1.5, and the detailed classification statistics are presented in Table 2.

### 2.2. Data Preprocessing

To improve the quality consistency of LIBS spectral data and enhance the input stability of the subsequent deep learning model, the raw spectral data were preprocessed in advance, mainly including multi-channel parsing and reconstruction, smoothing and denoising, normalization, and data augmentation during the training stage.

During the continuous online acquisition of LIBS spectra, two types of unavoidable invalid signal interference are present. On the one hand, because the spectrometer is typically activated before the laser cleaning process begins, a large number of pure background noise spectra without excitation features are inevitably collected during this time window. On the other hand, during the actual paint stripping process, weak signals lacking obvious characteristic elemental emission peaks may also be acquired due to random physical factors such as local surface morphology fluctuations of the sample, slight focal offset, or insufficient plasma excitation [31,32]. If these two types of anomalous spectra with low signal-to-noise ratios are directly input into the deep learning model, they will not only dilute the true material compositional features but also cause the network to learn invalid background noise distributions, thereby seriously interfering with the accurate classification of paint stripping states.

Therefore, a relative intensity threshold was introduced, using the maximum peak intensity of each individual spectrum over the full wavelength range as the evaluation criterion. Only when this maximum intensity exceeded 1000 a.u. was the spectrum identified as a valid spectrum containing effective excitation information. The threshold of 1000 a.u. was determined empirically based on preliminary inspection of the raw LIBS spectra acquired under the present experimental conditions. Spectra below this level generally corresponded either to background-only signals collected before laser irradiation or after laser shutdown, or to weak-emission spectra caused by insufficient plasma excitation, in which characteristic peaks could not be reliably distinguished from baseline fluctuations. Therefore, noise spectra and weak-signal spectra below this threshold were directly discarded. Here, the term “interference” refers to invalid spectral signals acquired during continuous online acquisition, rather than to a separately defined temporal parameter. After screening with this preprocessing procedure, 7–9 high-quality valid spectra could typically be retained within the same laser scanning sampling region. The retained spectra were then averaged point by point along the wavelength axis to generate a representative spectrum for that region, thereby improving the signal-to-noise ratio and the stability of data representation.

**Figure 6:**
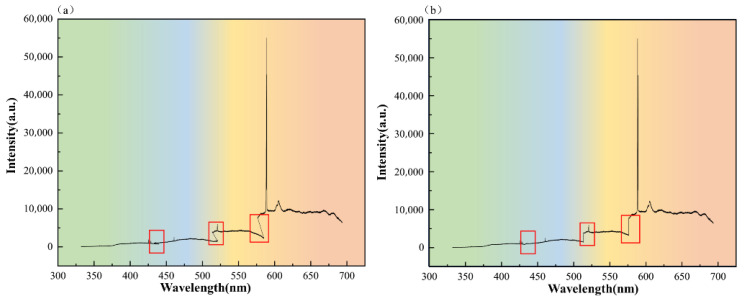
A schematic illustration of the multi-channel spectral reconstruction process: (**a**) raw multi-channel spectra; (**b**) reconstructed spectra.

Subsequently, the segmented spectra were sorted and concatenated according to the wavelength coverage of the four spectral channels to construct a complete one-dimensional spectral sequence. Owing to the physical beam-splitting characteristics of the multi-channel spectrometer, a certain degree of overlap exists between the end bands of adjacent channels. To reduce signal discontinuities at the stitching boundaries and avoid the local loss of critical characteristic peaks, a seamless stitching strategy was adopted in the overlapping bands by retaining the higher intensity value at each corresponding position. Finally, the reconstructed full spectrum was uniformly mapped by linear interpolation and aligned into a standard sequence with a fixed dimension of 4096, which was used as the direct input to the subsequent SE-ResNet1D network. The multi-channel spectral reconstruction process is shown in Figure 6.

On this basis, the reconstructed spectra were further subjected to smoothing, denoising, and normalization. Specifically, moving average filtering with a window size of 5 was applied during preprocessing to eliminate high-frequency spectral spikes. Subsequently, Z-score standardization and Min–Max normalization were performed in sequence to map all spectral features precisely into the range of 0–1, thereby eliminating the interference caused by absolute fluctuations in overall signal intensity. Meanwhile, to improve the model’s robustness against signal fluctuations and feature loss under practical operating conditions, composite random perturbation augmentation was applied to the spectral samples only during the training stage, including the addition of Gaussian white noise (standard deviation of 0.02), random amplitude scaling (0.9–1.1×), random shifting of the spectral sequence (±20 sampling points), and local random masking in which the intensities of 10–50 consecutive wavelength points were randomly set to zero.

For the visual modality, considering that the visual feature extraction branch in this study employed ImageNet-pretrained ResNet18 as the backbone network, all original surface images were uniformly resized to a fixed resolution of 224 × 224 pixels to strictly match the default receptive field scale and input dimension of the pretrained model. During model training, a series of data augmentation strategies were introduced for the image dataset to effectively suppress overfitting, including random resized cropping (scaling range: 0.7–1.0), horizontal and vertical flipping, random rotation (±30°), color jittering (brightness = 0.3, contrast = 0.3, saturation = 0.2, hue = 0.1), grayscale conversion with a probability of 10%, and Random Erasing with an erasing area ratio of 2–15%. In contrast, during evaluation on the validation and test sets, in order to reflect the true physical morphology, the images were only uniformly resized and conventionally normalized based on the standard mean and standard deviation of ImageNet, without applying any random transformations. The detailed parameter settings of the data augmentation and preprocessing procedures are summarized in Table 3.

### 2.3. Deep Learning Model Construction

In this study, a multimodal deep learning model for paint stripping state recognition, termed the Paint stripping Gated Multimodal Fusion Network (PGMF-Net), was developed using LIBS spectra and surface images as joint inputs. The overall model consists of a spectral feature extraction branch, an image feature extraction branch, a gated weighted fusion module, and a classifier. The spectral input is a one-dimensional sequence with a length of 4096, while the image input is uniformly resized to a 224 × 224 RGB image. The model outputs five classes, namely Tc, Tc-Pr, Pr, Pr-As, and As.

#### 2.3.1. Dual-Branch Feature Extraction Module

(1) Spectral Feature Extraction Based on SE-ResNet1D

LIBS spectral data are essentially one-dimensional sequences containing discrete elemental emission peaks and continuous background noise. Compared with fully connected networks, which tend to disrupt the local features of spectral lines, one-dimensional convolution can accurately capture the physical patterns around characteristic wavelength bands by leveraging local receptive fields, and can form higher-level spectroscopic representations through hierarchical stacking [21,33]. Meanwhile, considering that complex laser cleaning spectra are often subject to strong continuous background noise interference, the present branch incorporates the squeeze-and-excitation (SE) attention mechanism [34,35].

**Figure 7:**
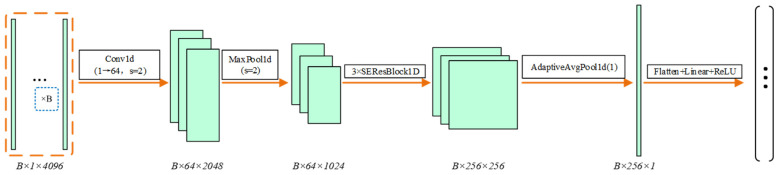
Flowchart of spectral feature extraction process.

Through adaptive recalibration, this mechanism automatically enhances variations in characteristic peaks associated with key elemental features, such as Ti in the topcoat, Cr in the primer, and Al in the substrate, while effectively suppressing irrelevant background-noise channels. Specifically, after the input spectral sequence passes through an initial one-dimensional convolution and max-pooling layer, it is successively mapped through three residual stages incorporating SE modules. The resulting features are then encoded into a 256-dimensional spectral feature vector via adaptive average pooling and a fully connected projection. The flowchart of spectral feature extraction is shown in Figure 7.

(2) Image feature extraction based on ResNet18

The recognition information conveyed by surface images during the paint stripping process is mainly reflected in two-dimensional features such as texture variations, edge transitions, and regional color differences. In this study, an ImageNet-pretrained ResNet18 was adopted as the backbone encoder for the image branch [36].

**Figure 8:**
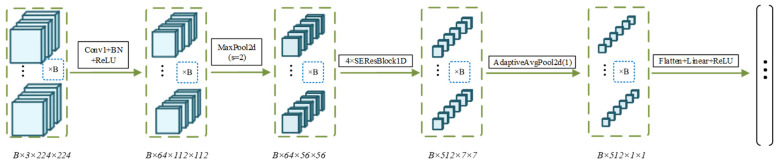
Flowchart of image feature extraction process.

Given the limited sample size in this study, comprising a total of 214 samples, a transfer learning strategy was introduced. Specifically, the initial layers and the first two residual stages of ResNet18 (layer1–2) were largely frozen to directly reuse the pretrained model’s capability for perceiving basic color patterns and geometric edges. Only the deeper layers, namely layer3 and layer4, were fine-tuned, so that the model could focus on learning high-level semantic features specific to the paint stripping task, such as residual coating and substrate gloss. Finally, the features were mapped into a 256-dimensional image representation vector $f_{i}$ through global average pooling and linear projection. This output is strictly aligned in dimensionality with the spectral branch, thereby laying the foundation for the subsequent adaptive fusion of heterogeneous features. The image feature extraction process is illustrated in Figure 8.

#### 2.3.2. Gated and Weighted Data Fusion Classification

To comprehensively exploit the high sensitivity of LIBS spectra to changes in material composition and the capability of surface images to characterize the evolution of macroscopic morphology, this study constructed a gated and weighted multimodal fusion classification module based on a dual-branch feature encoder. By introducing learnable control units, gated fusion dynamically adjusts the contribution of each modal feature to the final decision, thereby maintaining stable recognition performance even when modal quality is inconsistent or random noise interference is present, such as spectral defocusing or image occlusion [37].

At the feature fusion stage, the model first concatenates the spectral feature vector $f_{s}\in R^{256}$ and the image feature vector $f_{i}\in R^{256}$ in the feature dimension to obtain a joint feature representation $f_{\mathrm{cat}}\in R^{512}$. The concatenated feature is then passed through a gating network consisting of two fully connected layers. Specifically, the first layer reduces the feature dimension from 512 to 256 and is followed by a ReLU activation function, while the second layer projects the feature to a 2-dimensional weight vector corresponding to the two modalities. Finally, Softmax normalization is applied to obtain the adaptive fusion weights $w=[w_{s},w_{i}]$.

**Figure 9:**
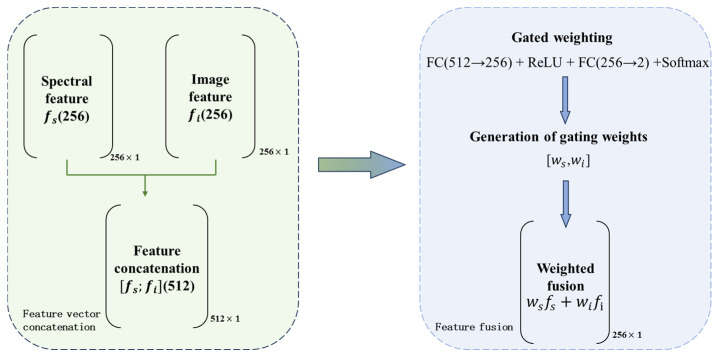
Flowchart of gated and weighted multimodal feature fusion.

Compared with fixed-weight fusion, this mechanism can identify the reliability of each modality in real time according to sample-level content. For example, when slight focal offset occurs during laser paint stripping and leads to weakened LIBS signals, the gating network can automatically reduce $w_{s}$ and increase $w_{i}$ through learning, thereby enabling the model to rely more on visual features for state discrimination and effectively alleviating the accuracy fluctuations caused by modal mismatch. Finally, $f_{m}$ is fed into the classifier to produce five-class output. The fusion process is illustrated in Figure 9.

## 3. Results and Analysis

### 3.1. Experimental Parameter Settings

The model developed in this study was implemented based on the PyTorch 3.12.0 framework, and the experimental hardware platform was configured with an Intel Core i7-9750H CPU and an NVIDIA GeForce GTX 1650 graphics card (4 GB VRAM). The multimodal dataset was randomly divided into the training, validation, and test sets in a strict ratio of 7:1.5:1.5. In terms of network initialization, the parameters of the fully connected layers in the gated fusion module were initialized using Kaiming Uniform Initialization, such that the multimodal gating weights were equally assigned to 0.5 at the beginning of training, thereby ensuring the unbiasedness and adaptive evolution of feature fusion. During model training, the AdamW optimizer was employed for parameter updating, with the weight decay coefficient set to $1\times 10^{-3}$ and the initial learning rate set to $3\times 10^{-4}$. To improve training stability and convergence quality, a learning rate scheduling strategy combining 5 epochs of linear warmup with cosine annealing was introduced. The loss function was chosen as cross-entropy loss with class weights and label smoothing (Label Smoothing, coefficient of 0.1) to jointly alleviate the risks of class imbalance and overfitting. In addition, the Mixup data augmentation strategy (α=0.3) was adopted to further improve the model’s robustness to input perturbations. The experiments were conducted with a batch size of 16 and a maximum of 100 training epochs, while an early stopping mechanism with a patience of 20 epochs was applied to ensure that the model weights with the best generalization performance were obtained.

### 3.2. Comparative Experimental Analysis

#### 3.2.1. Analysis of the Training Process and Convergence Comparison

**Figure 10:**
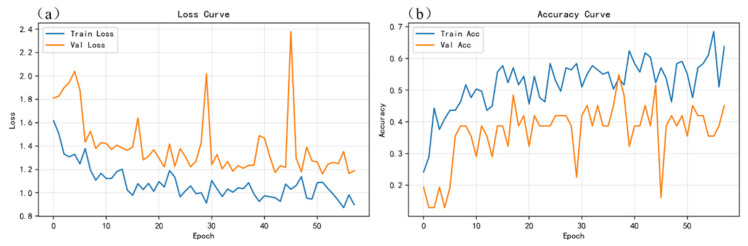
Curves of loss and accuracy variations during the training process of the spectral-only model: (**a**) loss curves for the training and validation sets; (**b**) accuracy curves for the training and validation sets.

**Figure 11:**
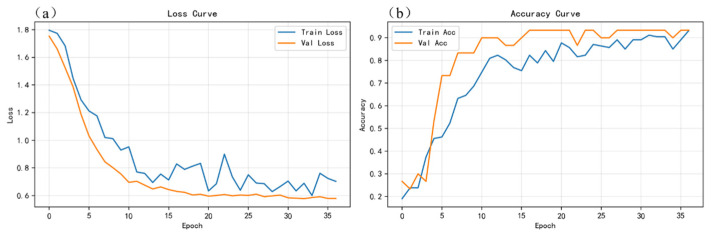
Curves of loss and accuracy variations during the training process of the image-only model: (**a**) loss curves for the training and validation sets; (**b**) accuracy curves for the training and validation sets.

**Figure 12:**
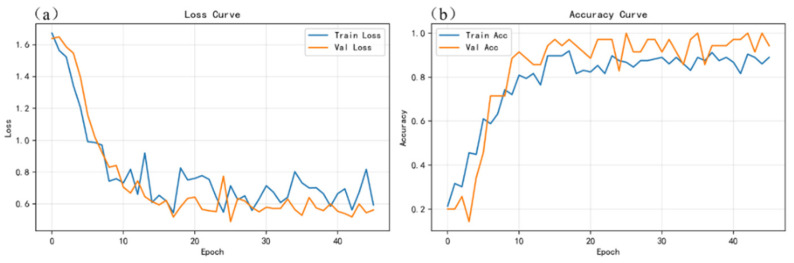
Curves of loss and accuracy variations during the training process of the multimodal model: (**a**) loss curves for the training and validation sets; (**b**) accuracy curves for the training and validation sets.

To verify the learning capability and training stability of the proposed gated and weighted multimodal fusion model (PGMF-Net), Figure 10, Figure 11 and Figure 12, respectively, present the loss and accuracy curves of the spectral-only (S), image-only (I), and multimodal (S+I) models over the entire training process.

From the training dynamics of the unimodal models, when relying solely on LIBS spectral input (Figure 10), the validation loss curve exhibits relatively pronounced fluctuations, and the validation accuracy remains at a low level. This is mainly because, in adjacent paint stripping stages, such as between slight residual coating and complete removal, the spectral compositional information from a single modality is highly susceptible to interference from local ablation fluctuations and substrate background noise, making it difficult for the model to establish clear classification boundaries on this basis. In contrast, the convergence behavior of the image-only model (Figure 11) is significantly improved, with both the training and validation losses decreasing smoothly and stabilizing at relatively low levels, indicating that surface morphology and two-dimensional color-difference information provide a more intuitive and stable representation of state evolution.

On this basis, the multimodal fusion model (Figure 12) exhibited the best convergence performance. Benefiting from the regularization strategies introduced in this study, such as Label Smoothing and Mixup, the training and validation curves of the multimodal model closely matched each other, with no obvious generalization gap, thereby effectively overcoming the overfitting problem that is prone to occur with small-sample datasets. More importantly, compared with the unimodal networks, the multimodal model achieved the fastest loss reduction, the highest validation accuracy, and the smallest fluctuations. This fully demonstrates that PGMF-Net successfully realizes the complementary integration of spectral and image information through the gated fusion mechanism, showing excellent feature learning efficiency and robustness during training, and thereby establishing a reliable foundation for the subsequent quantitative comparison of classification performance.

#### 3.2.2. Comparison of Experimental Results Between Unimodal and Multimodal Models

Furthermore, to quantitatively analyze the contributions of different input modality settings to paint stripping state recognition performance, comparative experiments were conducted under a unified experimental setting. Five model settings were considered: a unimodal model using only LIBS spectra as input (S), a unimodal model using only surface images as input (I), a late-fusion multimodal model, an early-fusion multimodal model, and the gated-fusion multimodal model proposed in this study (S+I, i.e., PGMF-Net). The quantitative evaluation results of these models on the test set are presented in Table 4.

The results in Table 4 intuitively reveal the representational characteristics and limitations of each modality. When only the spectral modality (S) was used, the overall recognition performance of the model was relatively limited, with an accuracy and Macro F1-score of 61.76% and 64.97%, respectively. This is mainly because, in adjacent paint stripping stages, such as between slight residual coating and complete removal, the differences in local plasma emission spectra are relatively small, making it difficult to independently achieve high-accuracy state discrimination.

In contrast, when only the image modality (I) was used, the model performance improved substantially, with the Accuracy and Macro F1-score increasing to 88.57% and 89.42%, respectively. This fully demonstrates that the two-dimensional spatial features contained in surface images, such as macroscopic texture, edge transitions, and regional color differences, possess strong representational capability for laser paint stripping states and serve as the dominant source of information for the discrimination process.

To further examine the effects of different multimodal fusion strategies, both late fusion and early fusion baselines were introduced for comparison. As shown in Table 4, the late fusion model achieved an accuracy of 87.88% and a Macro F1-score of 89.00%, which did not provide a clear improvement over the image-only model. This suggests that simply combining unimodal decisions at the final prediction stage is insufficient to fully exploit the complementary information between LIBS spectra and surface images. By contrast, the early fusion model achieved an accuracy of 90.91% and a Macro F1-score of 91.69%, outperforming both unimodal models. This result confirms that feature-level multimodal integration can more effectively combine the two information sources and improve recognition performance.

Among all compared models, the proposed S+I (PGMF-Net) achieved the best overall performance, with an accuracy of 94.12% and a Macro F1-score of 94.64%. Compared with the early fusion baseline, PGMF-Net further improved the accuracy by 3.21 percentage points and the Macro F1-score by 2.95 percentage points. These results indicate that LIBS spectra and surface images provide complementary compositional and morphological information, and that the proposed gated fusion strategy can better exploit such complementarity under the current experimental setting, thereby improving the recognition of laser paint stripping states.

#### 3.2.3. Comparative Analysis of the Confusion Matrix and Category-Specific Errors

**Figure 13:**
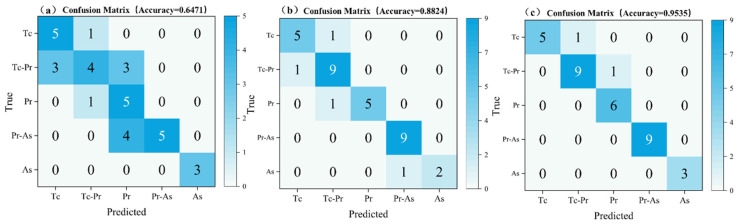
Confusion matrices: (**a**) spectral-only model; (**b**) image-only model; (**c**) multimodal fusion model.

Although Table 4 has already shown from the perspective of overall metrics that the multimodal fusion model achieves better recognition performance, comprehensive evaluation metrics alone cannot reveal the specific misclassification patterns among different categories. To this end, Figure 13 further presents the confusion matrices under the three modality settings.

As shown in Figure 13a, the spectral-only model achieved acceptable recognition performance for the two states with extreme physical characteristics, namely pure topcoat (Tc) and substrate damage (As), whereas its severe misclassifications were concentrated in the intermediate transition stages of the paint stripping process. Specifically, three Tc-Pr samples were misclassified as Tc, and another three Tc-Pr samples were misclassified as Pr; meanwhile, four Pr–As samples were misclassified as Pr. The physical reason for this phenomenon lies in the fact that, during the dynamic transition period of laser stripping of multilayer coatings, plasma excitation usually contains mixed interfacial elements. In particular, when the system is in the Tc-Pr stage, if the exposed primer area at the laser spot is very small, its LIBS spectral response becomes highly similar to that of the pure topcoat, and the single spectral sequence is therefore highly susceptible to insufficient local sampling representativeness, leading to severe cross-state confusion.

Figure 13b shows that the diagonal elements of the confusion matrix for the image-only model are clearly more concentrated, and its overall recognition accuracy is significantly higher than that of the spectral model. This indicates that, by virtue of its macroscopic receptive field, the image modality can more stably capture two-dimensional texture features such as mottled coating residues and regional color differences, thereby effectively alleviating the misclassifications caused by local sampling fluctuations. Nevertheless, the image-only model still exhibits a small number of errors between adjacent states, with one Tc-Pr sample misclassified as Tc and one As sample misclassified as Pr-As. Such errors tend to occur at microscopic physical critical points where the coating thickness is extremely thin but has not yet been completely removed, at which point the differences in surface color and texture have become exceedingly subtle and pure visual features have reached their representational limit.

**Figure 14:**
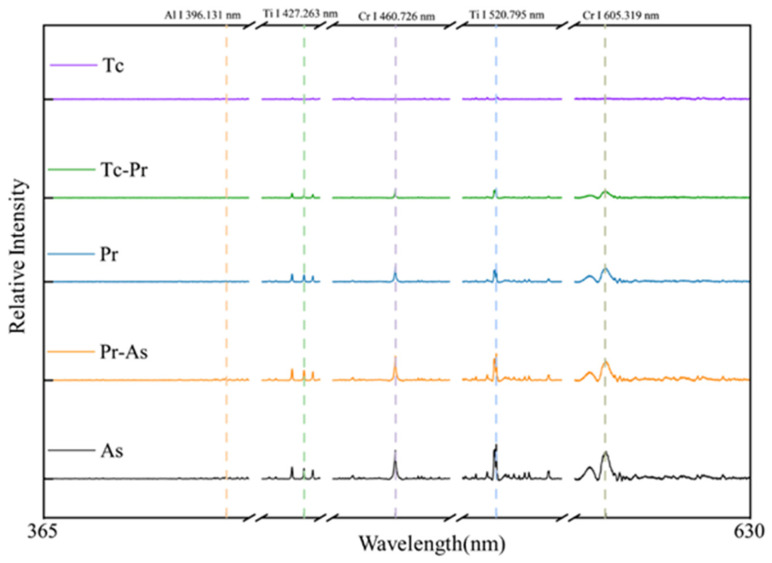
LIBS spectra under different states.

To further explain the sources of misclassification at the boundaries between adjacent states, the responses of key characteristic emission peaks in representative LIBS spectra under different states were extracted and jointly analyzed together with the model recognition results, as shown in Figure 14.

As can be seen from Figure 14, the overall characteristic peaks are relatively weak in the Tc state, with only a weak sharp peak visible at Ti I 520.795 nm. When the state changes to Tc-Pr or Pr, Cr I 460.726 nm and Cr I 605.319 nm gradually emerge and intensify, indicating that primer-related information has already been incorporated into the spectra. In the As state, Al I 396.131 nm begins to appear, suggesting that the laser action has further involved the aluminum alloy substrate. Meanwhile, Ti I 520.795 nm does not disappear during the state transition process, but instead exhibits an increasing trend. This is mainly because, as the laser power increases, material ablation and plasma excitation are enhanced, thereby increasing the relative intensity of the Ti element. Therefore, the recorded spectra reflect the combined effects of multiple material layers during the cleaning process, which is also an important reason why the single-spectrum model in Figure 13a is more prone to recognition confusion.

### 3.3. Model Validation

To further reduce the influence of randomness introduced by a single data split, stratified five-fold cross-validation [38] was performed on the spectral-only model, the image-only model, and the multimodal fusion model PGMF-Net, and the results are presented in Table 5. It can be seen that the performance differences among the models under different modality settings are consistent with those obtained in the previous fixed-split experiments, namely that the multimodal model achieves the best overall performance, followed by the image-only model, while the spectral-only model remains relatively weaker.

**Figure 15:**
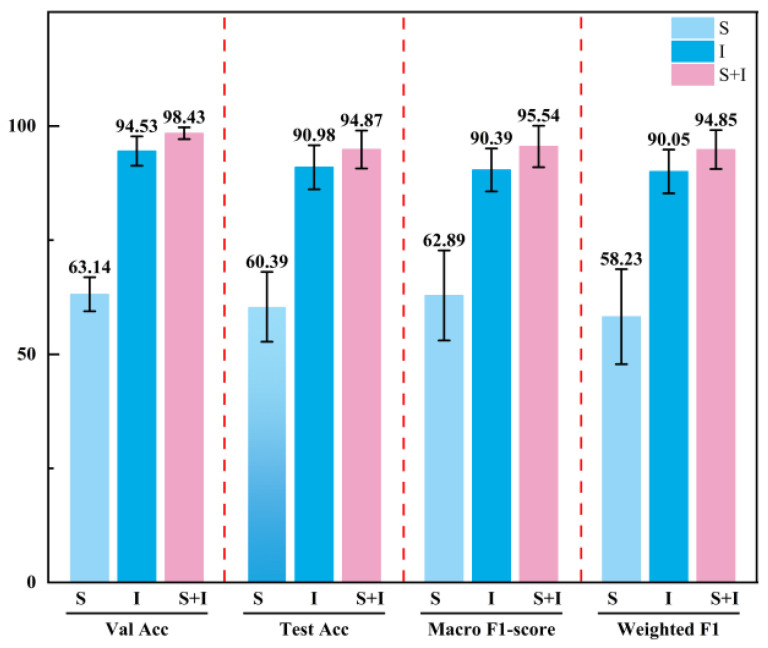
Comparison of the average performance and standard deviations of models with different modality settings under five-fold cross-validation.

To more intuitively illustrate the average performance of models with different modality settings and their fluctuation ranges under five-fold cross-validation, a bar chart was plotted, as shown in Figure 15. In this figure, the height of each bar represents the mean value of the corresponding evaluation metric across the five experiments, while the error bar denotes the corresponding standard deviation. As can be seen from the figure, the multimodal fusion model these results suggest that the two modalities provide complementary information in terms of validation accuracy, test accuracy, Macro F1-score, and Weighted F1, and the overall error bars were relatively short. This indicates that, even when faced with different data subset partitions, the multimodal fusion model still exhibited excellent consistency in feature learning and stability in results, effectively overcoming the performance fluctuations caused by distribution differences in small-sample data.

From the perspective of average performance, the multimodal fusion model outperformed both unimodal models in validation accuracy, test accuracy, Macro F1-score, and Weighted F1, indicating that the joint modeling of spectral and image information can effectively enhance the model’s overall capability to recognize the five cleaning states. In particular, compared with the image-only model, PGMF-Net improved the average test accuracy from 90.98% to 94.87%, representing an increase of 3.89%. The Macro F1-score increased from 90.39% to 95.54%, corresponding to an improvement of 5.15%. The Weighted F1-score increased from 90.05% to 94.85%, corresponding to an improvement of 4.80%. Because Macro F1-score assigns equal weight to each category, these results indicate that the multimodal fusion strategy not only improves the overall classification accuracy, but also enhances the model’s comprehensive recognition capability across all categories.

Further analysis from the perspective of standard deviation shows that the multimodal fusion model exhibited relatively small fluctuations across all four metrics, with standard deviations of 1.28%, 4.15%, 4.55%, and 4.26% for validation accuracy, test accuracy, Macro F1-score, and Weighted F1, respectively. Compared with the multimodal model, the image-only model showed slightly larger fluctuations in most metrics, although the overall difference was small, whereas the spectral-only model exhibited the most pronounced variability, indicating that it was more sensitive to the partitioning of the training samples. Overall, PGMF-Net not only achieved higher average recognition performance, but also demonstrated better result consistency and generalization capability across different folds.

**Figure 16:**
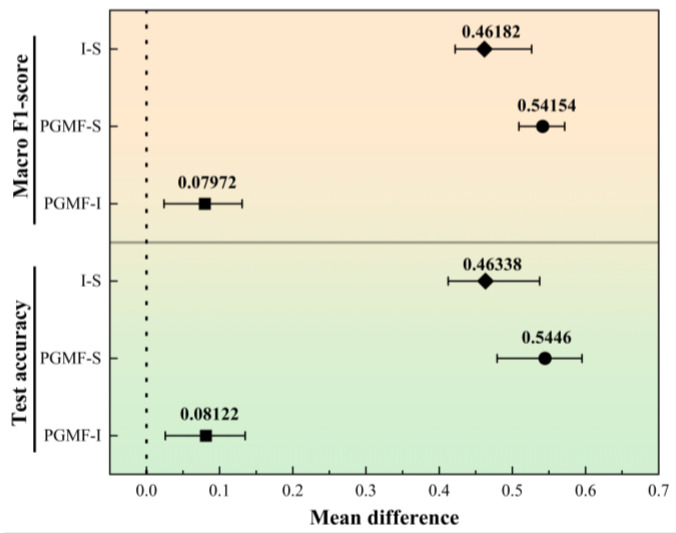
Mean performance differences with 95% bootstrap confidence intervals for pairwise modality comparisons.

To further complement the statistical comparison among different modality settings, a paired sample-level bootstrap analysis was additionally performed based on the prediction results. The mean differences and their 95% bootstrap confidence intervals for test accuracy and Macro F1-score are presented in Figure 16. As shown, all pairwise mean differences remained positive, and the corresponding confidence intervals did not cross zero, indicating that the observed performance advantages were directionally stable under repeated resampling rather than being caused by random fluctuations. Specifically, the multimodal model exhibited the largest positive difference relative to the spectrum-only model, while it also maintained a positive improvement over the image-only model. Meanwhile, the image-only model consistently outperformed the spectrum-only baseline. These results further support the effectiveness and stability of the proposed multimodal fusion strategy for paint stripping state recognition.

In summary, the results of the stratified five-fold cross-validation, together with the paired bootstrap analysis, further verify the effectiveness and stability of the proposed multimodal fusion model for the current laser paint stripping state recognition task. Compared with the unimodal methods, PGMF-Net not only achieves superior average performance, but also maintains a stable positive advantage under different data partitioning and repeated resampling conditions. These findings provide more reliable support for the subsequent development of online monitoring and decision-making models. Nevertheless, it should be noted that the present validation is still based on the current dataset and process setting. For transition states such as Tc-Pr and Pr-As, as well as the As damage state, their generalization capability under a broader range of process parameters still needs to be further examined through future expansion of the sample size and parameter space.

### 3.4. Experimental Validation

**Figure 17:**
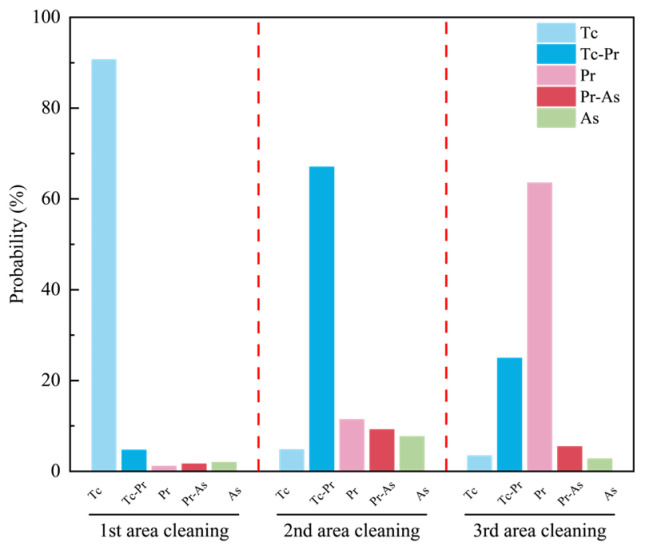
Classification probability distributions of the cleaned regions under different laser powers.

**Figure 18:**
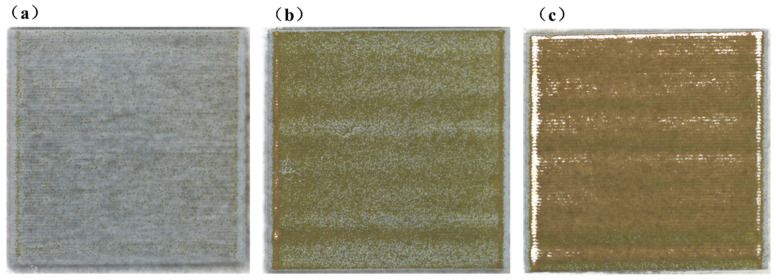
Macroscopic morphologies of the cleaned regions under different laser powers: (**a**) 1st area cleaning (400 W); (**b**) 2nd area cleaning (500 W); (**c**) 3rd area cleaning (600 W).

To validate the practical applicability of the constructed multimodal recognition model, experimental verification was carried out using samples coated with a bilayer coating system. The sample surface was sequentially coated with an approximately 60 μm white polyurethane topcoat (Tc) and an approximately 30 μm green epoxy primer (Pr) (see Section 2.1). The laser parameters were set as a repetition frequency of 12 kHz, a scanning speed of 3000 mm/s, a scanning area of 20 mm × 20 mm, and laser powers of 400 W, 500 W, and 600 W, resulting in a total of three experimental groups. These parameters were selected based on preliminary experiments to ensure that the cleaning process remained in a stable and relatively uniform operating condition. Since the recognition task investigated in this study was defined under the prerequisite of uniform paint removal, parameters such as scan speed, repetition frequency, incidence angle, and focal offset were kept constant, while laser power was selected as the only varying factor to drive the progressive evolution of paint stripping states for model validation. The state probability distributions of the three cleaned regions are shown in Figure 17, and the corresponding macroscopic morphologies are presented in Figure 18.

The results indicate that, with increasing laser power, the dominant state of the cleaned region evolves sequentially from Tc to Tc-Pr and then to Pr. Specifically, under the 400 W condition, the sample surface appeared overall gray-white, with continuous topcoat coverage and no obvious primer exposure, and the model output was dominated by Tc (90.66%), which was consistent with the image characterization, indicating correct classification of this region. Under the 500 W condition, scattered Tc residues were observed on the sample surface, while the green Pr began to emerge, showing typical interlayer transition characteristics, and the model output was dominated by Tc-Pr (67.02%), which agreed well with the coexistence of topcoat residues and primer exposure in the image, indicating correct classification of this region. Under the 600 W condition, the sample surface exhibited an overall yellow-green appearance, indicating that the white topcoat had been largely removed and that the green primer had become the dominant exposed layer, and the model output was dominated by Pr (63.52%), which was consistent with the macroscopic morphology, indicating correct classification of this region. In summary, the dominant categories output by the model correspond well to the actual surface morphologies of the cleaned regions under different laser powers and can effectively reflect the state evolution during laser paint stripping from topcoat retention to topcoat–primer transition and finally to primer exposure.

## 4. Conclusions

This study developed a multimodal state recognition method for aircraft skin laser paint stripping by fusing LIBS spectra and surface images to identify five states: topcoat (Tc), topcoat–primer transition (Tc-Pr), primer (Pr), primer–substrate transition (Pr-As), and substrate damage (As). The results showed that the proposed PGMF-Net outperformed the unimodal, early-fusion, and late-fusion models, achieving an accuracy of 94.12% and a Macro F1-score of 94.64% on the test set, with an average accuracy of 94.87% under stratified five-fold cross-validation. By integrating compositional and morphological information, the proposed method improved recognition performance for complex paint stripping states and showed good agreement with the actual surface changes under different laser powers. Overall, this study provides a feasible technical basis for online state recognition and subsequent process feedback in aircraft skin laser paint stripping.

## Figures and Tables

**Table 1:** Basic parameters of spectrometer.

Model	Spectral Range	Resolution	Integration Time
BZ2000	336–436 nm	0.15 nm	1 ms–60 s
	435–517 nm	0.15 nm	1 ms–60 s
BZ4000	516–583 nm	0.16 nm	1 ms–60 s
	580–700 nm	0.20 nm	1 ms–60 s

**Table 2:** Dataset split results.

Category	Training Set	Validation Set	Test Set	Total
Tc	22	5	5	32
Tc-Pr	42	9	9	60
Pr	30	6	6	42
Pr-As	42	9	9	60
As	14	3	3	20

**Table 3:** Detailed parameter settings of data augmentation and preprocessing.

Modality	Method	Parameter Setting	Applied to
Spectral	Gaussian white noise	std = 0.02	Training set only
	Random amplitude scaling	0.9–1.1×	Training set only
	Random wavelength shift	±20 sampling points	Training set only
	Random masking	10–50 consecutive wavelength points set to zero	Training set only
Image	Random resized crop	scale = 0.7–1.0	Training set only
	Random horizontal flip	*p* = 0.5	Training set only
	Random vertical flip	*p* = 0.5	Training set only
	Random rotation	±30°	Training set only
	Color jitter	brightness = 0.3, contrast = 0.3, saturation = 0.2, hue = 0.1	Training set only
	Random grayscale	*p* = 0.1	Training set only
	Random Erasing	*p* = 0.2, area ratio = 2–15%	Training set only
	Deterministic resize + normalization	resize to 224 × 224; ImageNet mean/std normalization	Validation and test sets

**Table 4:** Classification performance under different modality settings on the test set.

Modality	Accuracy/%	Macro Precision/%	Macro Recall/%	Macro F1-Score/%
S	61.76	66.95	70.22	64.97
I	88.57	91.03	88.95	89.42
S+I (Late fusion)	87.88	89.33	88.89	89.00
S+I (Early fusion)	90.91	92.67	91.11	91.69
S+I (PGMF-Net)	94.12	95.14	94.67	94.64

**Table 5:** Cross-validation performance under different modality settings (mean ± standard deviation).

Modality	Accuracy/%	Macro Precision/%	Macro Recall/%	Macro F1-Score/%
S	63.14 ± 3.73	60.39 ± 7.65	62.89 ± 9.86	58.23 ± 10.41
I	94.53 ± 3.22	90.98 ± 4.83	90.39 ± 4.70	90.05 ± 4.80
S+I (PGMF-Net)	98.43 ± 1.28	94.87 ± 4.15	95.54 ± 4.55	94.85 ± 4.26

## Data Availability

The original contributions presented in this study are included in the article. Further inquiries can be directed to the corresponding author.

## Abbreviations

The following abbreviations are used in this manuscript:

PGMF-Net	Proposed gated and weighted multimodal fusion network
Tc	Topcoat
Tc-Pr	Topcoat–primer transition
Pr	Primer
Pr-As	Primer–substrate transition
As	Substrate damage
